# Campus landscape types and pro-social behavioral mediators in the psychological recovery of college students

**DOI:** 10.3389/fpsyg.2024.1341990

**Published:** 2024-09-04

**Authors:** Yi Xu, Tingting Wang, Jinsong Wang, Hongtao Tian, Ruixin Zhang, Yixuan Chen, Hong Chen

**Affiliations:** School of Art, Anhui University, Hefei, China

**Keywords:** campus landscape types, psychological recovery, pro-social behavior, mediating roles, perception restorative

## Abstract

**Introduction:**

Physical and mental health problems of college students are becoming more prominent, and contact with nature has a positive effect on physical and mental health. This paper investigates the psychological recovery effect of different types of campus green space landscape on college students. From the perspective of college students’ perception of campus landscape types, the green space, blue space, gray space and movement space of three universities in Anhui Province are investigated.

**Methods:**

Through choose campus landscape types and questionnaires, structural equation modeling (SEM) and mediation modeling were constructed on the role of college students’ perception of campus landscape types on psychological recovery.

**Results:**

It was found that the level of landscape type perception had a significant effect on the effect of psychological recovery and the generation of pro-social behavior, with no significant gender difference, while psychological recovery also had a positive effect on the generation of pro-social behavior. The study also found that campus landscape type not only directly affect students psychological recovery, but also promote psychological recovery through the mediating role of pro-social behavior.

**Discussion:**

The study reveals the effects of campus landscape type on college students’ psychological recovery, and pro vides a basis for planning campus of different types.

## 1 Introduction

As college students are in the specific transition stage of life growth, they are facing the pressure of employment, economy and emotion, and inevitably produce a variety of psychological problems, such as loneliness, depression, social anxiety, emotional crisis, neurasthenia and so on (Xinbin and Shoucai, 2006). The Chinese national mental health development report (2021 ~ 2022) blue book shows that about 21.48% of college students may have a risk of depression, 45.28% of college students may have anxiety risk, shown in the blue book in the 18–24 this age group corresponding to the average depression scale and anxiety scale is the highest in the whole population age, this stage is the college students age (Xiaolan F. et al., 2023). For college students in Hefei, the total mental health literacy rate is 29.63%, and there are more serious psychological problems among college students (Xiaoyan L. et al., 2023). In the past, research on college students’ mental health has mostly focused on stress, personality, and coping, ignoring the important factor of an individual’s attitude toward others and society. Pro-social behavior is often taken as an important measure of an individual’s ability to actively adapt to society (Zhengguang, 2020). Pro-social behavior refers to positive attitudes and actions such as support, caring, helping and giving toward the behavior of others or society, which are spontaneous for the purpose of meeting the needs of others or society, and enhancing the well-being of others or society (Hay, 1994). Pro-social behaviors are positive, beneficial and specific behaviors that can lead to cognitive development, enhance emotional experiences, strengthen interpersonal interactions and interpersonal cooperation, and promote social relationships and social adaptation (Xinbin and Shoucai, 2006; Guo, 2017). For college students whose mental health problems are becoming more and more prominent, pro-social behaviors can alleviate college students’ stress and anxiety through specific behaviors, improve their sense of well-being and subjective well-being (Layous et al., 2017), and positive emotions can reduce the physiological and psychological effects of negative emotions (Taylor et al., 2017), which ultimately contributes to the development of students’ mental health.

## 2 Research basis

### 2.1 Potential mechanisms linking green space to pro-social behavior, mental health

There is a close relationship between pro-environmental and pro-social behaviors, and studies have shown that exposure to natural environments can promote pro-social behaviors (Markevych et al., 2017). Green environments can provide a comfortable, pleasant, and relaxing environment that enhances people’s mental health status and social skills by relieving stress and improving mood, which in turn leads to a greater willingness to give and share (Hoffman, 2018). Therefore, people in green environments are more likely to trigger positive pro-social behaviors such as donating, sharing, volunteering, and aiding (Dunfield, 2014; Hoffman, 2018; Aram et al., 2019). There are potential pathways linking green spaces to health, pro-social behaviors through three dimensions: harm mitigation, capacity building and resilience (Markevych et al., 2017). Green spaces can achieve the linkage pathway of harm mitigation by mitigating pollution and reducing environmental stressors that negatively affect the development of pro-social behaviors. Secondly for capacity building, green space promotes physical activity and social interaction and is more likely to provide opportunities for co-operation and trust building in interactions (Abrams et al., 2015). In terms of resilience, Stress Recovery Theory and Attention Recovery Theory can be effective in promoting and restoring physical and mental health (Kaplan, 1995). High-frequency exposure to green spaces generates positive emotions, and positive factors are a key component of subjective well-being, effectively promoting pro-social behaviors through increased life satisfaction (Jia et al., 2014).

### 2.2 Campus landscape perception type and college students’ pro-social behavior, psychological recovery

Both stress recovery theory (SRT) (Ulrich, 1983) and attention recovery theory (ART) (Kaplan, 1995) suggest that attention recovery and emotion regulation are effective in natural environments. Frequent exposure to nature is beneficial to human health and well-being. Short-term exposure to forests, city parks, gardens, and other natural environments can reduce stress and depressive symptoms, restore attention fatigue, and improve physical and mental health (Deng et al., 2020). Campus green spaces are closely linked to the daily lives of college students, and exposure to green spaces for school students can promote psychological recovery. According to Zhang’s ecological and sociological study, the higher the landscape preference and activity preference scores, the better the effect of the environment on the physical and mental recovery of college students (Tian, n.d.). Different campus landscapes have different recovery effects on college students. Chang et al. (2018) investigated college students in three universities in Beijing and found that campus green space visiting behavior had a moderating effect on college students’ emotions. Visiting campus green spaces significantly increased positive emotions, significantly decreased negative emotions, and resulted in better cognitive recovery (Wang et al., 2020). Experiments have shown that college students’ blood pressure decreased significantly in all outdoor green space landscapes, with systolic blood pressure decreasing tens of times more in woodland landscapes than indoors, and that woodland landscapes provide the greatest stress recovery benefits for college students (Jian and Shufen, 2021). In the last 2 years, studies have found that water landscapes have a better relaxation effect on college students, and water landscapes can provide college students with stronger feelings of remoteness and glamor, followed by woodlands (Xing, 2018). Xing (2018) found that under normal mental stress conditions, the higher the vegetation coverage, the more the campus landscape with natural barge water bodies is conducive to mental stress recovery. Blue spaces on campus help to recover from mental fatigue, while gray spaces promote mental recovery more through motor behavior, and their features of effective communication and companionship can increase the potential for recovery (McIntyre et al., 1990). Studies have confirmed that short stays on the plaza, walks or meditation can significantly improve mood and concentration. Open gray spaces on campus such as commuter spaces and athletic spaces can provide varying degrees of recovery, and physical activity may be a more important stress reliever than an unsurprising outdoor landscape (Herranz-Pascual et al., 2019).

Dyment’s study showed that green schoolyards promote social inclusion and that students become more civilized, tolerant, cooperative and beneficial to teacher-student interactions. At the same time, the diversity of green school environments provides conditions for a variety of activities that reduce bullying and other aggressive behaviors (Dyment and Bell, 2008). Campus water feature areas have the ability to regulate climate, enhance physical and mental health, and guide emotions and behaviors, which can positively influence pro-social behaviors and may also ameliorate behaviors that are detrimental to the environment (Alcock et al., 2020). By participating in the maintenance and protection of water features, university students develop awareness and responsibility for protecting the environment, which leads to good social behaviors. Du et al. (2022) found that students prefer to exercise in the gray space, believing that the gray space is more attractive and more likely to produce worship and obsession. Campus landscapes make it easier to generate goodwill behaviors and a sense of social responsibility, and promote communication and mutual support among university students, thus enhancing cohesion and social ties (Puhakka, 2021).

In previous studies, more research has explored the important role played by the quantity and quality of park green space in influencing children’s health and pro-social behaviors (McEachan et al., 2018; Putra et al., 2020, 2021, 2022), as well as exploring the relationship between factors such as frequency of visits, perceived crowding and perceived beauty of park green space and pro-social behaviors (Noël et al., 2021; Noël and Dardenne, 2022). Existing research evidence on the type of campus green space and students’ pro-social behavior and mental health is scarce. This paper aims to remedy this lack of evidence by attempting to use pro-social behavior as a mediating variable to establish a mediation model between campus landscape perception, college students’ mental health and pro-sociality, and to explore the effects of pro-social behavior and type of campus landscape on college students’ psychological recovery. Specifically, the hypotheses of this study are proposed:

*H1*. The perception of campus landscape type of college students has a positive predictive effect on prosocial behavior;

*H2*. College students' perception of campus landscape type has a positive predictive effect on restoring mental health;

*H3*. The prosocial behaviors of college students in different types of landscapes may be predictive of mental health.

*H4*. That college students' pro-social behavior mediates the relationship between campus landscape type perception and perception restorative.

## 3 Research methodology and data collection

### 3.1 Research objectives

#### 3.1.1 Research area

**Figure 1:**
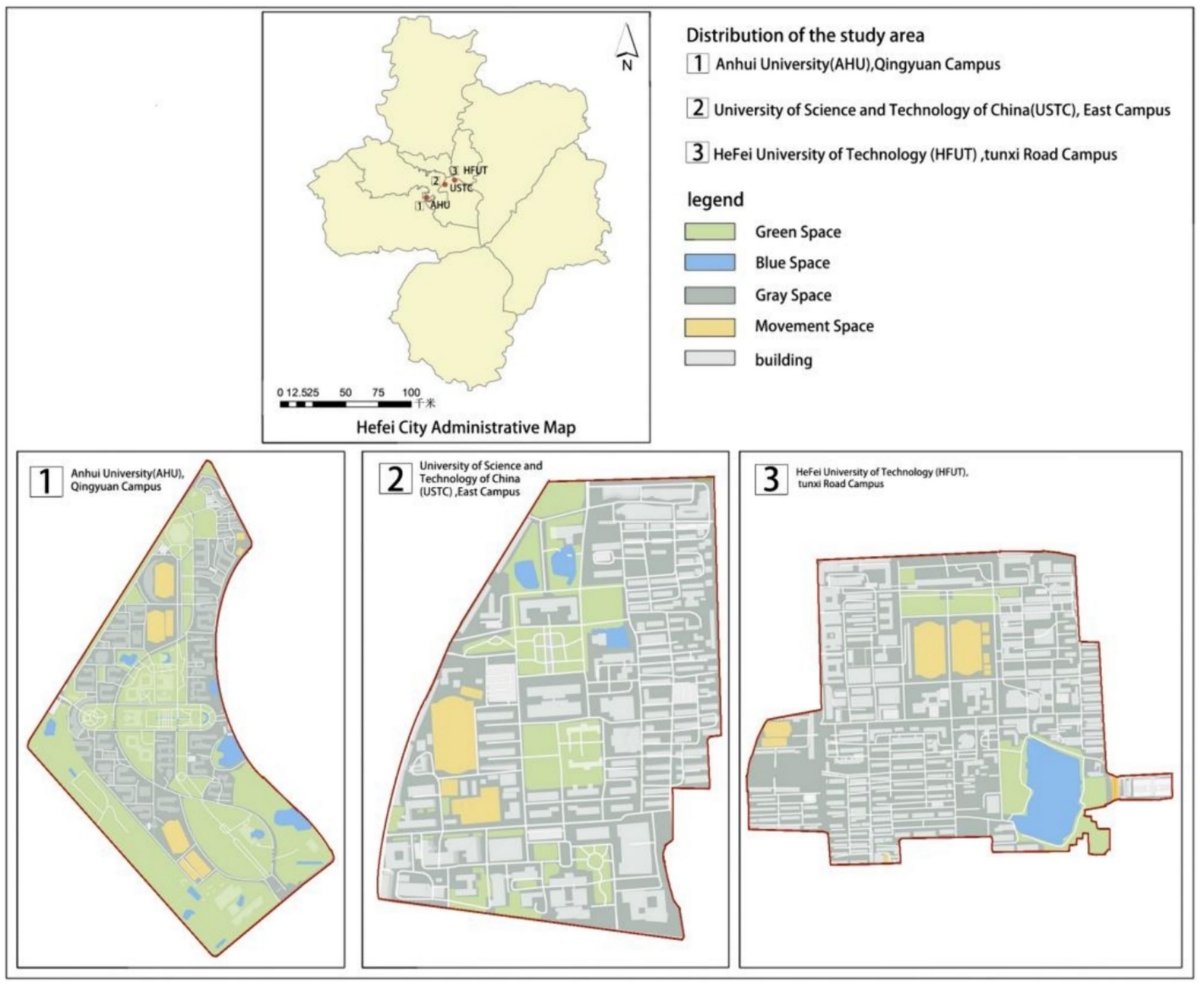
Research area and floor plan.

Three schools, Anhui University Chingyuan Campus, University of Science and Technology of China (USTC) East Campus, and Hefei University of Technology (HFUT) Tunxi Road Campus in Hefei City, Anhui Province, were used as the research area. Among them, Anhui University Chingyuan Campus was built the latest and has the largest proportion of public green space, followed by the East Campus of University of Science and Technology of China, and Hefei University of Technology Tunxi Road Campus was built the earliest and has the smallest proportion of public green space (Figure 1).

Photographs were taken of four different landscape types: green space, blue space, gray space, and movement space in the three campuses, and 12 photographs were selected as samples for visual stimulation, as shown in Table 1.

**Table 1:** Environment and characteristics within the research campuses.

Typology	Sample photos	Characterization
Green space	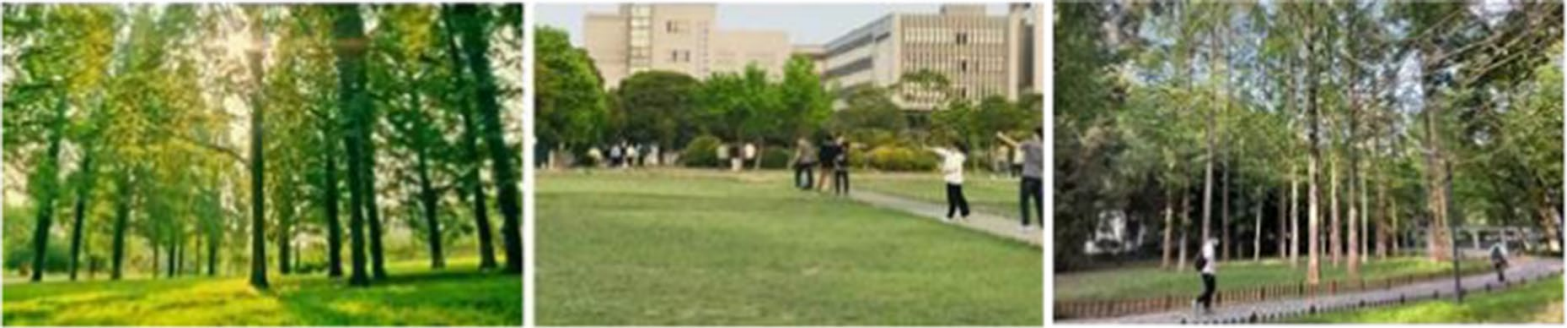	Over 75% green coverage, dominated by large lawns and tall trees.
Blue space	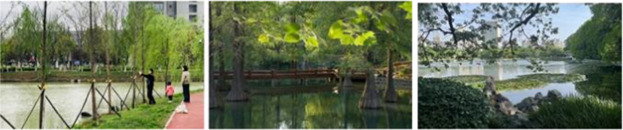	The landscape is mainly open water, accompanied by some plantings and waterfront facilities, and is relatively quiet.
Gray space	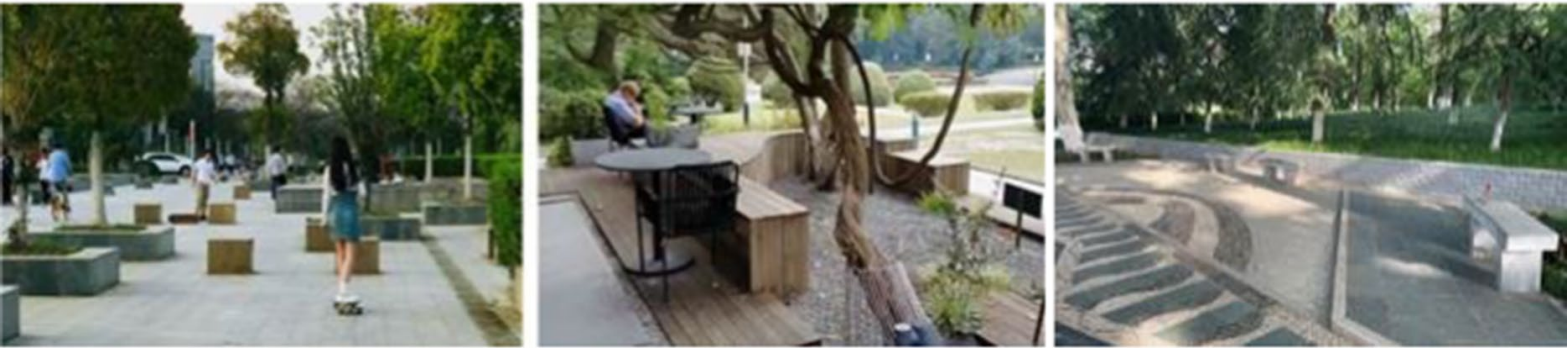	Predominantly hard-paved sidewalks with more resting seating and a small amount of plantings.
Movement space	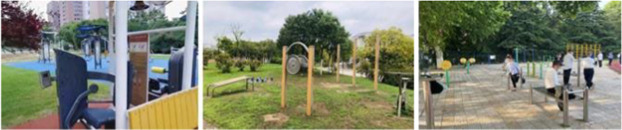	The sports facilities are varied and are mainly lawn with a few trees.

### 3.2 Participants

The study was carried out on Questionnaire Star, an online platform for Anhui Province college students, including both undergraduates and postgraduates. All participants are voluntary and can withdraw from the questionnaire at any time. For some sensitive topics, a no-contact, remote web-based questionnaire was used, which allowed respondents to answer questions more naturally and truthfully without the presence of an investigator (Si-Xi, 2022). Participation was voluntary and participants could withdraw from the survey at any time. A total of 308 questionnaires were completed; however, after eliminating non-college students who rarely or never visited green spaces and those aged 35 years and above, 264 questionnaires remained, making the final validity rate 85.7%.

The questionnaire survey reveals that the majority of respondents were college students aged 18–35. Male participants accounted for 27.7% whereas female participants comprised 72.3% of the sample. Specifically, 89.4% of participants were between the ages of 18–25, with 26–35 year olds accounting for 8%. In terms of majors, 45.8% were from liberal arts programs followed by 33% from science and engineering programs, and 21.2% from arts programs (Table 2).

**Table 2:** Description of the distribution of sample characteristics.

Variables	Options	Frequency	Percentage
Age	Under 18 years old	7	2.70%
	18–25 years old	236	89.40%
	26–35 years old	21	8.00%
Sex	Male	73	27.70%
	Female	191	72.30%
Specialty	Science and Engineering	87	33.00
	Liberal Arts	121	45.80%
	Art	56	21.20%

### 3.3 Data collection

In order to investigate the relationship between college students and campus landscape type perception, perception restorative, and pro-social behavior, the study asked participants to complete an online questionnaire after viewing the sample photos. The questionnaire consisted of four parts: the first part obtained basic information (basic information, green space type preference, and frequency and sociability), the second part aimed to inform the university students about the types and characteristics of the campus landscapes in the photographs by evaluating their perceptions and preferences of the landscape types in terms of the four dimensions of peacefulness, naturalness, spatiality, and sociability using a five-point Likert scale (ranging from 1 = strongly disagree to 5 = strongly agree) (Grahn and Stigsdotter, 2010). The third part used the Partial Perceived Restorative Scale (PRS) revised by Zhang-Zhan et al. (2008) to investigate the psychological recovery in campus landscapes at school universities, which includes four dimensions of remoteness, extensibility, charming, and compatibility, and the original scale consisted of a total of 18 items, of which 12 items were selected to investigate the psychological recovery in campus landscapes at school universities, and the 12 items were investigated using a five-point Likert scale (ranging from 1 = strongly disagree to 5 = strongly agree); and the fourth part based on the characteristics of college students’ activities in campus green space, this paper selected the three characteristics of social reciprocity, public labor and social morality in college students’ pro-social behaviors as summarized by Xinbin and Shoucai (2006) to assess the potential pro-social behaviors of college students in campus landscapes (Table 3).

**Table 3:** Questionnaire survey scale section.

Scale	Dimension (math.)	Dimensionality interpretation	Question item
Green space perception Scale	Peacefulness	Peaceful, undisturbed, clean and cozy.	1. It is quiet and cozy. 2. It is not disturbed by too many people.
	Naturalness	A wild, nature-like environment where flora and fauna grow naturally and where there is a rich diversity of plants, animals and insects.	1. There is a sense of being in the midst of nature. 2. There is a rich diversity of plants, animals and insects.
	Spatiality	Includes both openness and seclusion, with open and coherent spaces where you can move around freely, and closed spaces with a certain degree of seclusion.	1. There are spaces made up of plants where people can move around. 2. It is relatively safe, private, and enclosed, so you can watch others play or move around on your own.
	Social	People can gather here for chats, societies, sports or other recreational activities.	1. It is a place for socializing, such as eating, chatting, playing sports and having fun. 2. There is a strong campus culture here.
Perceived restorative scale	Remoteness	Feeling able to escape from a state of fatigue or stress.	1. Being here can bring me a feeling of being away from the hustle and bustle. 2. The environment here can help me relax my tight mood. 3. I do not feel constrained in my study and daily life here.
	Extensibility	The space has enough content for people to feel that they are escaping from stress.	1. The environment here is in harmony with the surroundings. 2. The environment here can make me extend memories or associations. 3. The scenery is well designed.
	Charming	The environment is perceived as interesting and captures people’s attention.	1. The environment here has attractive qualities. 2. There is much to explore and discover here. 3. I would want to spend more time doing activities here.
	Compatibility	Surroundings are consistent with people’s preferences and activities.	1. I can do the things I like to do here. 2. I can get a sense of belonging here. 3. I can adapt to the environment here very quickly.
Pro-social behavior	Social reciprocity	Giving benefits to the other party while also receiving benefits from the other party.	1. I prefer to confide in each other’s emotions with friends in a natural setting. 2. I would be willing to co-operate with others in a natural environment to complete an activity. 3. I would be more willing to help people in need in a natural environment.
	Public labor	Participation in public service, unpaid labor.	1. I would be willing to take an active part in the waste separation campaign organized by the school. 2. I am willing to complete public welfare practical labor (e.g., planting trees, spreading knowledge, etc.) in the natural environment. 3. I am more willing to join school clubs and organizations that carry out activities in the natural environment.
	Social morality	A code of behavior agreed upon by people living in a society for the benefit of the group.	1. I will not litter in the natural environment. 2. I will consciously maintain the public facilities on campus. 3. I will consciously follow the school’s environmental guidelines.

### 3.4 Statistical analysis

The data gathered underwent statistical analysis using SPSS 26.0. Initially, ANOVA was conducted to uncover significant variances between college students’ perception of the campus landscape type and perception restorative. Subsequently, structural equation modeling was employed to further examine the relationship between students’ perceived preference for unconventional campus landscapes, their perception restorative, and their pro-social behaviors. Finally, a mediation analysis was carried out using PROCESS 4.1 to examine the correlation between landscape type preference, psychological restoration, and pro-social behavior level.

## 4 Analysis of research results

### 4.1 Reliability and validity

The questionnaire design of this paper includes four parts: basic personal information, green space perception scale, partial perception restorative scale and improved pro-social behavior scale. SPSS 26.0 software was used to test the internal reliability of 30 items in the four parts of the questionnaire, and the results are shown in Table 4, in which the Cronbach’s alpha coefficient is 0.924, which is much higher than 0.5, indicating that the data are highly reliable (Eisinga et al., 2013). The structural validity of the questionnaire was tested by exploratory factor analysis, and the validity of the questionnaire was tested by KMO and Bartlett’s spherical test, and the results are shown in Table 5, with a coefficient of 0.915 for the KMO test (Gim Chung et al., 2004). Meanwhile, according to the spherical test, the significance of this test is infinitely close to 0, which rejects the original hypothesis, so the questionnaire has good validity in this case.

**Table 4:** Reliability test Cronbach alpha coefficients.

Reliability statistics		
Cronbach alpha	Cronbach alpha based on normalized terms	Item count (of a consignment etc.)
0.924	0.924	30

**Table 5:** Results of KMO and Bartlett’s sphere test.

KMO quantity of sample suitability		0.915
Bartlett’s test of sphericity	Approximate cardinality	4012.536
	Degrees of freedom	406
	Significance	0.000

### 4.2 Common method bias test

When collecting data through self-report methods, it is possible that common method bias may exist. As a precaution, the collected data underwent testing for common method bias using Harman’s one-way test. The unrotated exploratory factor structure identified 5 eigenvalues greater than 1, with the first factor explaining 34.494% of the variance (<40%). Therefore, this study does not exhibit any significant common method bias.

### 4.3 Descriptive statistics and analysis

The third section of the survey examined how college students evaluated the restorability of various landscape types shown in the photographs. Table 6 data suggests that the restorative scores of all four types of on-campus landscapes exceeded 3, implying positive restorative effects for most college students. Of the spaces evaluated, the green space obtained the highest score (3.963 ± 0.585), followed by the blue space (3.790 ± 0.555) and the gray space (3.632 ± 0.878). In contrast, the movement space gained the lowest score (3.556 ± 0.729).

**Table 6:** Analysis of restorative potential of different landscape types.

Landscape type	*N*	*M*	*SD*	*SE*	95% C.I.	
					LL	UL
Green space	156	3.963	0.585	0.047	3.870	4.055
Blue space	71	3.790	0.555	0.066	3.658	3.921
Gray space	19	3.632	0.878	0.201	3.208	4.055
Movement space	18	3.556	0.729	0.172	3.193	3.918
Total	264	3.865	0.623	0.038	3.789	3.940

**Figure 2:**
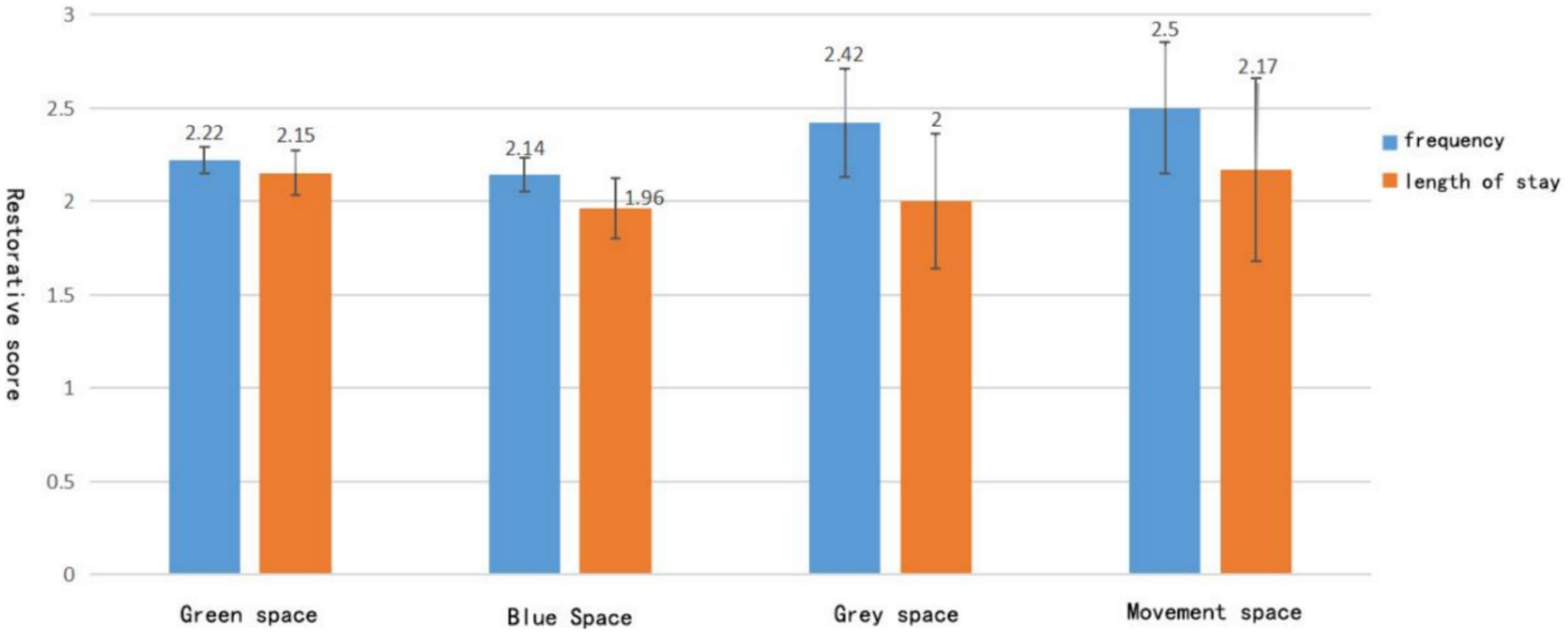
Frequency and duration of visits to different landscape types.

The examination of the frequency of green space visits among university students are less likely to actively visit green spaces. In particular, 79.2% of students visit green spaces only once or twice a week, and a mere 2.3% do so more than five times weekly, typically staying for under an hour. The majority of university students opt to visit green spaces, which accounts for 59.1% of visits, followed by blue spaces at 26.9%, with gray spaces accounting for 7.2% and sports spaces at 6.8%. According to the ANOVA results, Figure 2 illustrates how sports spaces have the highest visit frequency (2.5 ± 0.699) and the longest stay duration (2.17 ± 0.789), whereas blue spaces have the lowest visit frequency (2.14 ± 0.481) and the shortest stay duration (1.96 ± 0.848). College students engage in a variety of activities, with green spaces being mainly visited for relaxation (190 occurrences), recreation (86 occurrences), sports (34 occurrences), in addition to the pressure of study.

### 4.4 Correlation analysis

Exploratory analysis of the correlation between the variables through Pearson Correlation analysis, the greater the correlation coefficient *r* between two variables, the greater the correlation between them. This study discusses the correlation between campus landscape type perceptions of peacefulness, naturalness, spatiality, and sociality, perception restorative of remoteness, extensibility, charming, compatibility, pro-social social reciprocity, public labor, and social morality as shown in Table 7, and from the results, it can be seen that the dimensional scores of 11 variables are at a high level. The mean scores are all at a high level, and the correlation coefficients *r* between the variables of green space perception of peacefulness, naturalness, spatiality, and sociality, perception restorative of remoteness, extensibility, charming, compatibility, pro-social social reciprocity, public labor, and social morality are all greater than 0, with the corresponding minimum significance levels *p*-values less than 0.01, and the correlation coefficient *r* is greater than 0,indicating a significant positive correlation among all dimension variables in this analysis.

**Table 7:** Pearson correlation analysis between dimensions.

Dimensionality	Peacefulness	Naturalness	Spatiality	Social	Remoteness	Extensibility	Charming	Compatibility	Social Reciprocity	Public labor	Social virtual
Peacefulness	1										
Naturalness	0.714^**^	1									
Spatiality	0.707^**^	0.660^**^	1								
Social	0.601^**^	0.588^**^	0.702^**^	1							
Remoteness	0.413^**^	0.370^**^	0.397^**^	0.381^**^	1						
Extensibility	0.332^**^	0.289^**^	0.368^**^	0.413^**^	0.670^**^	1					
Charming	0.327^**^	0.315^**^	0.360^**^	0.258^**^	0.643^**^	0.645^**^	1				
Compatibility	0.327^**^	0.291^**^	0.364^**^	0.278^**^	0.635^**^	0.626^**^	0.711^**^	1			
Social reciprocity	0.222^**^	0.178^**^	0.258^**^	0.309^**^	0.475^**^	0.554^**^	0.515^**^	0.474^**^	1		
Public labor	0.152^*^	0.149^*^	0.236^**^	0.284^**^	0.406^**^	0.502^**^	0.410^**^	0.472^**^	0.598^**^	1	
Social morality	0.295^**^	0.215^**^	0.274^**^	0.222^**^	0.476^**^	0.448^**^	0.449^**^	0.362^**^	0.406^**^	0.392^**^	1

**At the 0.01 level (two-tailed), the correlation is significant; *Significant at the 0.05 level (two-tailed).

### 4.5 Path analysis

#### 4.5.1 SEM model analysis

**Figure 3:**
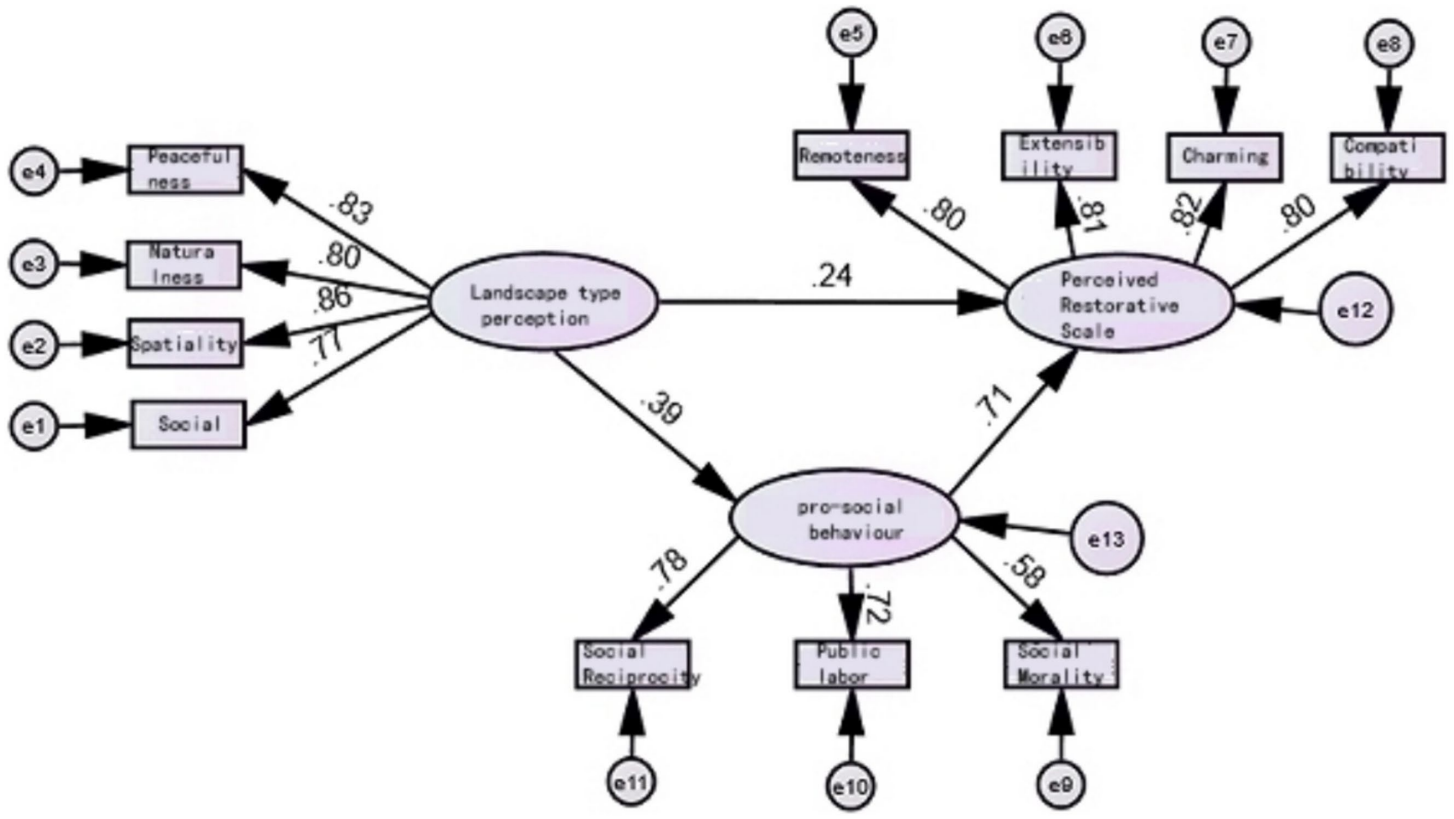
SEM model diagram.

AMOS26 was used to construct a structural equation model to validate and analyze Hypothesis H1, Hypothesis H2 and Hypothesis H3. The model includes three research variables: landscape type perception, perception restorative and pro-social behavior, and the structural model is shown in Figure 3.

#### 4.5.2 SEM model path relationship hypothesis test results

Through the results of path relationship analysis, it can be concluded that in the path hypothesis relationship test of this study, perception of landscape type significantly positively predicts Pro-social behavior (*β* = 0.39, *p* < 0.001), so hypothesis H1 is established; secondly, perception of landscape type has a significant positive predictive effect on perception restorative (*β* = 0.241, *p* < 0.001), so hypothesis H2 is established; Finally pro-social behavior was equally significant as a positive predictor of perception restorative (*β* = 0.715, *p* < 0.001), thus hypothesis H3 holds (Table 8).

**Table 8:** Results of the SEM path relationship test for each variable.

	Pathway relationships		Estimate	*S.E.*	C.R.	*P*
Pro-social behavior	←	Landscape type perception	0.382	0.08	4.751	***
Perception restorative	←	Landscape type perception	0.241	0.088	4.039	***
Perception restorative	←	Pro-social behavior	0.715	0.146	7.392	***

***p < 0.001.

### 4.6 Test of the mediating effect of college students’ landscape type preference on perception restorative

In order to test Hypothesis 4, that is, pro-social behavior mediates the psychological recovery process of college students in campus landscape type perception, the mediating effect test was conducted by using the non-parametric percentile Bootstrap method of bias correction, and 5,000 self-sampling tests using Model4 of the PROCESS macros in SPSS (Model4 is a simple mediation model). Perception of landscape type was set as the independent variable (X), perception restorative was set as the dependent variable (Y), as well as pro-social behavior was set as the mediator variable (M), and descriptive statistics and correlation analyses were first conducted for each variable as shown in Table 9.

**Table 9:** Descriptive statistics and correlation of each variable of the mediator model.

	*M*	*SD*	Landscape type perception	Perception restorative	Pro-social behavior
Landscape type perception	31	5.79	1		
Perception restorative	46.38	7.481	0.462***	1	
Pro-social behavior	35.23	5.531	0.333***	0.666***	1

***p < 0.001.

According to the results of the mediating effect test of the regression method (Table 10), it can be seen that there is a significant relationship between perception restorative on the perception of landscape type in the test of model 1 (*β* = 0.597, *p* < 0.001), indicating that the total effect is established. In the test of Model 2 there was a significant relationship for the mediating variable (*β* = 0.318, *p* < 0.001), while in the test of Model 3 there was a significant effect of perception restorative on the perception of landscape type (*β* = 0.349, *p* < 0.001) and a significant effect of pro-social behavior on perception restorative (*β* = 0.780, *p* < 0.001).

**Table 10:** Regression analysis of the relationship of variables in the mediation model.

Models	Model 1		Model 2		Model 3	
Variables	Perception restorative		Pro-social behavior		Perception restorative	
Indicators	*β*	*t*	*β*	*t*	*β*	*t*
Perceived landscape type	0.597	8.436***	0.318	5.715***	0.349	5.876***
Pro-social behavior					0.780	12.529***
*R*	0.462		0.333		0.713	
*R*-sq	0.214		0.111		0.509	
*F*	71.171***		32.661***		135.251***	

***p < 0.001.

**Figure 4:**
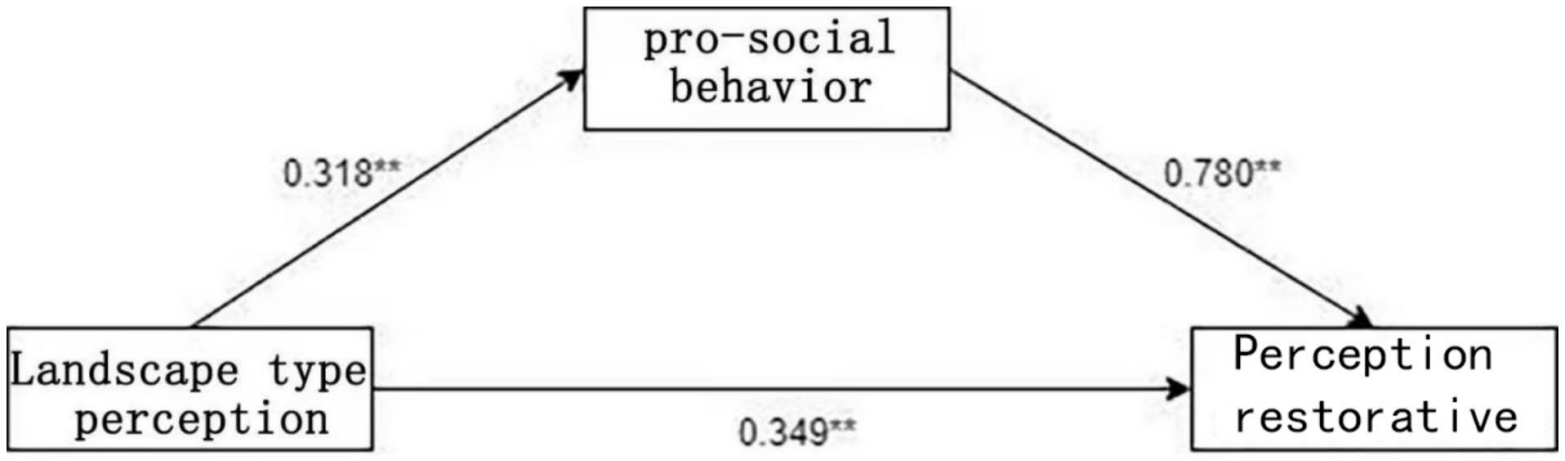
Intermediation pathway diagram.

Bootstrap95%CI of the mediating effect does not contain 0 ([0.138–0.37]), indicating that the mediating role of pro-social behavior in the model is established, and the mediating path diagram is shown in Figure 4.Therefore, the pro-social behavior of college students partially mediates the level of mental health recovery. According to the results of coefficient test, the direct effect accounted for 58.49%, and the indirect effect accounted for 41.51%, and the results verify hypothesis H4 (Table 11).

**Table 11:** Analysis of mediating effect of pro-social behavior.

Effect relationship	Efficacy value	LLCI	ULCI	Efficiency ratio
Total effect	0.597	0.458	0.737	
Direct effect	0.349	0.232	0.466	58.49%
Indirect effect	0.248	0.138	0.375	41.51%

## 5 Discussion and suggestions

This study investigates the association between the type of campus landscape perceived by college students, their perception restorative, pro-social behavior and psychological recovery. The research utilizes college students from three universities based in Anhui as participants. The findings confirm the substantial interplay between college students’ perception of campus landscape type, perception restorative and pro-social behavior and pro-social behavior mediates psychological recovery across various campus landscape types. This article represents a valuable addition to the current research, offering a vital resource for campus landscape planning. Furthermore, it can provide significant assistance in the psychological recuperation of university students.

### 5.1 Analysis of the psychological recovery potential of campus landscape types

The study discovered that the university’s green space environment can affect the physical and mental health and aid in stress recovery of university students through measurements of landscape type preference perception and perception restorative. Table 6 presents the analysis of different landscape types and their restorative potential. The results indicate that most college students perceived green space, blue space, gray space, and sports space as having positive restorative effects. However, for school students, the restorative potential of various campus landscape types differed. Green space (3.963 ± 0.585) and blue space (3.790 ± 0.555) had higher mean scores compared to gray space (3.632 ± 0.878) and sports space (3.556 ± 0.729).

The blue-green space contains abundant natural resources and is the preferred campus landscape for contemporary university students. Research in environmental psychology has demonstrated that exposure to natural environments results in reduced daily stimuli (Berman et al., 2008), mitigates physiological and emotional stress, and enhances recovery from cognitive fatigue and concentration caused by daily stress (van den Berg et al., 2010). However, based on the survey findings, college attendees least frequently visited the blue area (2.14 ± 0.481) and stayed for the shortest duration (1.96 ± 0.848). Based on the feedback gathered from participants’ questionnaires, the primary reasons for college students’ visits to the blue space were relaxation and viewing. However, unlike urban parks, college campuses lack the necessary infrastructure for students to rest and move around, which could explain the shorter duration of use.

The exercise space scores the lowest in restorative potential (3.556 ± 0.729). Nevertheless, as shown in Figure 2 ANOVA results, this area has the highest visit frequency (2.5 ± 0.699) and longest stay duration (2.17 ± 0.789). Both frequency and duration of exercise visits reveal positive restorative effects for college students (Markevych et al., 2017). The campus is the primary location for college students to reside and engage in leisure activities, the green space on campus possesses significant ecological value, as well as providing an opportunity for physical exercise. This has been demonstrated in numerous studies that outdoor exercise environments often have greater recuperative quality than indoor alternatives, with potential antidepressant and anxiolytic effects (Barton and Pretty, 2010).

The restorative potential of gray space (3.632 ± 0.878) and the moderate scores of frequency of visit (2.5 ± 0.699) and duration of use (2.17 ± 0.789) indicate that gray space also has a positive restorative potential and can promote the recovery of attention. In contrast to Du Y’s findings (Du et al., 2022), this study also found that gray space is not only charismatic but also malleable. Since gray space is more widely distributed and accessible on campus, gray space is used more frequently. It has been shown that gray spaces characterized by effective communication and companionship can increase the potential for recovery (McIntyre et al., 1990). According to participant feedback, free social activities such as club activities, competitions, and chats are available in gray space, and these may stimulate the recovery potential of gray space and promote stress and mental recovery among college students.

### 5.2 The effect of campus landscape type perception on perception restorative and pro-social behavior

The study found a significant correlation between college students’ perception of campus landscape types, perception restorative and pro-social behavior. Regression analysis showed that there was a positive and significant effect between college students’ perceptions of different campus landscape types and perception restorative, i.e., the higher the level of perceptions of landscape types, the better the effect on psychological recovery. This finding shows that although individual college students perceive different campus landscape types differently, they all have significant restorative effects, which is consistent with Guo et al. (2023). Specifically, the peacefulness characteristic in landscape space has the highest degree of correlation with the remoteness characteristic. It can be shown that the more obvious the calmness and relaxation characteristics of a spatial environment, the more effective it is to get away from the hustle and bustle and to promote relaxation. This may be the reason why college students are more willing to choose a quiet, gentle and comfortable landscape environment to release pressure and relax when they are busy with their studies and anxious. In addition, there is a correlation between the dimensions of sociality and extensiveness. This is due to the fact that the public service facilities in the landscape space can prompt college students to engage in social activities, thus exerting the characteristics of sociality and extensibility in the landscape space and transferring the psychological pressure of college students.

Positive and significant effect between perception of campus landscape type and pro-social behavior, i.e., the higher the level of perception of landscape type, the more likely pro-social behavior. Preferences for different landscape types have different effects on pro-social behavior. Green space often involves landscape maintenance and environmental protection issues, frequent visitors to green space will be more conscious of avoiding pollution of the environment, more likely to produce environmental awareness and behavior; sports space is not only a fitness and exercise place, but also an important social interaction social place, in the process of exercise through interaction and communication to produce a sense of closeness and trust, showing social and pro-social behavior; sports space with social characteristics, Gray space and other campus landscapes with social characteristics usually create a relaxing and pleasant atmosphere, which can promote communication and interaction between individuals, thus promoting social reciprocal behaviors such as college students making friends, sharing experiences, and establishing social connections. In addition, people in the landscape space will also pay more attention to the protection and utilization of the natural environment and humanistic landscape, and through the interactive experience with nature and culture, they will generate a sense of concern and responsibility for the environment and society, which is also a form of social reciprocity.

### 5.3 Pro-social behavior has a mediating role

**Figure 5:**
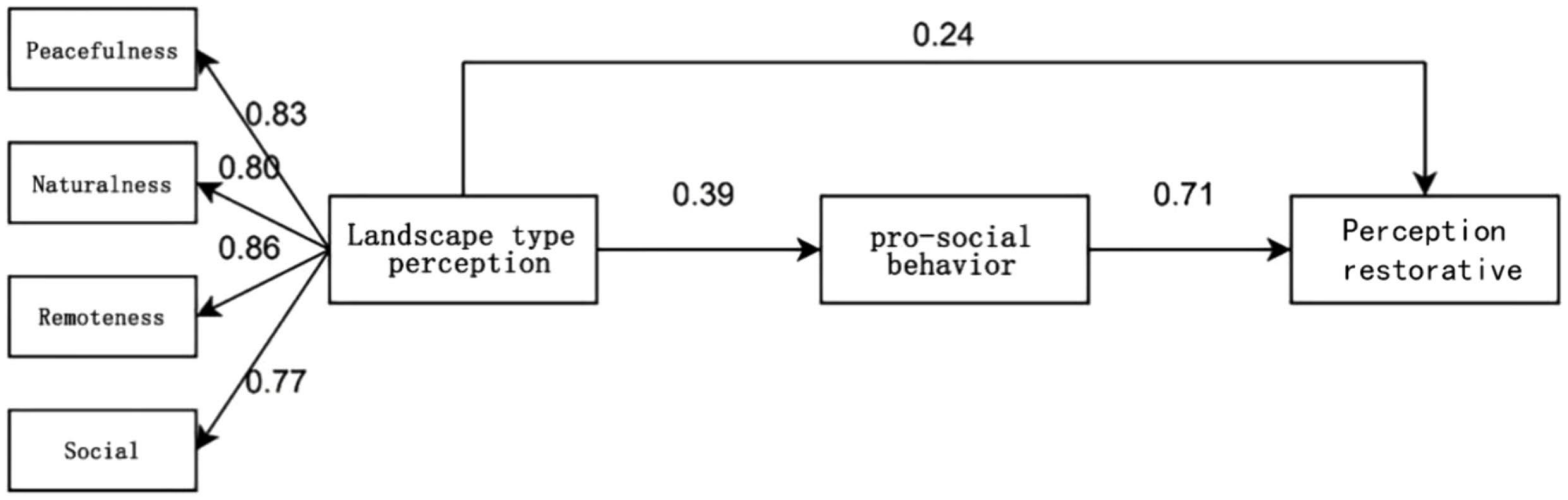
Effects of pro-social behavior.

The results of the study showed that college students’ pro-social behavior had a partial mediating role in the perception of landscape type and perception restorative. The total effect was 0.597, the direct effect was 0.349, and the indirect effect was 0.248, accounting for 41.51% of the total effect. Perceptions of campus landscapes affect college students’ emotions and attitudes, and stimulate pro-social behaviors. The theory of “warm-glow giving” states that when an individual feels that he or she has succeeded in helping the people around him or her, a sense of happiness and satisfaction will be generated, which also has a certain effect on alleviating negative emotions such as depression, anxiety and stress (Andreoni, 1989). Most people have the so-called “warmth” emotion, and the altruistic behaviors of individuals will produce good feelings, and the power of warmth can induce altruistic behaviors. Surveys show that college students are more willing to help others, share information, and have a stronger sense of environmental protection in the campus landscape environment. When college students perceive a high-quality, comfortable and pleasant landscape space, they will be more willing to communicate and share with people around them and help others, and these pro-social behaviors can make college students feel relaxed and enhance their sense of well-being, which has the effect of promoting psychological recovery. This is consistent with Lazar L’s findings that implementing pro-social behaviors has a positive effect on reducing the psychological and physiological responses to stress (Aknin et al., 2019; Guo et al., 2023) (Figure 5).

### 5.4 Recommendations for campus landscape planning

This research study found that the perception of campus landscape type and the psychological recovery and pro-social behavior of college students can positively influence each other. The campus environment is the main place for teachers and students to study, live and socialize, and optimizing the design of the campus landscape plays an important role in promoting the generation of pro-social behaviors among college students, relieving their psychological pressure, and facilitating the recovery of their attention. Therefore, it is recommended to consider the following points in campus landscape planning and design:

(1) Improve accessibility and utilization of all landscape types on campus. In this study, it was found that the percentage of college students who never or hardly visit green spaces was as high as 13.6%. College students accounted for as high as 13.6%, and compared with green space, the access rate of blue space, gray space and sports space is even lower, which may be due to the fact that the campus landscape space does not meet the actual needs of college students well. It is suggested that the campus landscape space should be reasonably arranged to optimize the compatibility between behavioral activities, and the settings of different campus landscape types should be integrated into the routes of college students’ learning and living behaviors, so as to match the motivation and behavioral characteristics of college students’ participation in all kinds of campus activities, and to better satisfy college students’ functional needs for various landscape types. For example, the pavement, benches and resting platforms of squares and passages can be combined with the creation of study and club activities to conceal the space boundaries; in the design and planning of sports space, not only can the sports venues satisfy the sports needs of college students, but also leisure facilities (such as fitness facilities, badminton courts, etc.) can be placed in the unused space around the buildings to provide college students with more diversified and convenient sports activity opportunities. Opportunities. In addition, increasing the configuration of facilities in the public space, providing a sufficient number of rest seats and seats with natural views in the campus public space, and creating a sense of visual aesthetics, art, and space through art and design techniques to enhance the charm and attractiveness of the campus landscape can significantly increase the duration and frequency of college students’ stay in the campus landscape (Lazar and Eisenberger, 2022).
(2) Emphasize the psychological restorative function of each landscape type on campus. Increasing academic and employment pressure affects the psychological health of college students. Rest facilities in public space play a role in the psychological recovery of college students. An appropriate amount of rest facilities should be provided in all types of landscape space on campus to meet the needs of college students for relaxation, socialization and rest. Reasonable configuration of the number of activity facilities on campus and the compatibility of activity areas can promote the generation of leisure activities and social behaviors of college students, helping them to relieve psychological pressure and restore their attention. The openness of the lawn should be enhanced in the blue-green space to reduce the obstruction of the line of sight, and the green space can choose some medicinal plants with mood-calming effects, such as jasmine, lavender, violet, and evening primrose, etc. By configuring plants with restorative functions, the landscape space can play a role in stabilizing mood and relieving stress in a relaxing and natural atmosphere. Gray space is mainly dominated by hard ground, so adding flowers, shrubs and other visually rich plants in the space, reasonably configuring plant communities, and creating a comfortable spatial environment can effectively achieve the role of improving and soothing emotions. It is also possible to combine the psychological debugging and relaxation of college students with plant and animal conservation activities, such as setting up healing gardens in suitable places on campus, stimulating the visual, tactile, olfactory, auditory and gustatory senses of college students through plant and animal conservation activities to relieve stress, get rid of numbness and fatigue, and regulate disordered physiological functions. In addition, different colors have different impacts on individual’s mood and mental state, and the reasonable use of different plants and building materials can not only bring a sense of visual balance and break the monotony of a single color, but also play a positive role in the recovery of college students’ mood and stress.
(3) Increase the setup arrangement of diversified interaction space and content in campus landscape planning. This paper proves through research that pro-social behavior plays an intermediary role in college students’ campus landscape type perception and psychological recovery, and social activities are one of the important ways. Social activities are more likely to stimulate college students’ empathy and sympathy, which help them understand the situation and needs of others, and then show more care, concern and support, and promote the development of pro-social behavior. High-quality landscape environment helps to enhance students’ environmental awareness and ecological literacy, prompts college students to adopt a more environmentally friendly and sustainable lifestyle, helps to cultivate students’ sense of social responsibility, and enhances their active participation in environmental protection. In campus landscape planning, natural elements and topographical conditions can be used to provide informal interaction sites in blue-green spaces with high visiting frequency, creating diversified social conditions, helping college students to establish intimate and trusting social relationships through communication, mutual assistance and support, and laying a solid foundation for pro-social behaviors such as cooperation and reciprocity. Schools can guide college students to develop pro-environmental awareness and behaviors such as protecting the environment and treating animals kindly through the construction of experiential practices, environmental and food education, and horticulture courses, and promote the development of moral character such as physical and mental health, self-esteem and self-confidence; cultivate a positive attitude toward the environment and empathy among students; and increase the knowledge of food and form healthy eating habits. Schools can also make full use of abandoned land to grow edible plants by relying on the natural and cultural resources and infrastructure of the campus, and organize related activities through student clubs. Students can put forward their own suggestions and ideas for the campus edible landscape, and participate in the planning, planting, cultivation, harvesting, and food preparation and sharing, taking into account the functional value of edible plants. The whole process will enhance the emotional identity of the students and their sense of belonging and attachment to the university, thus generating positive emotions such as happiness, joy and fulfillment.

## 6 Conclusion

Starting from the perception of campus landscape types by current college students, based on the field research of Anhui University Chimeyuan Campus, Hefei University of Technology Tunxi Road Campus and University of Science and Technology of China as well as the analysis of spatial characteristics, structural equation modeling (SEM) and regression model to analyze and verify the path and effect of the perception of campus landscape type on the psychological recovery effect. The main conclusions are as follows.

First, different campus landscape types (green space, blue space, gray space, and movement space) had good restorative effects, with no significant differences by gender. Second, there was a significant correlation between college students’ perceptions of campus landscape types and perception restorative and pro-social behaviors, with spatial characteristics having the greatest effect on restorative effects, followed by peacefulness. Meanwhile, pro-social behavior also had a significant effect on perception restorative, with the social reciprocity dimension having the most significant effect. Last but not least, the study confirmed that the perception of campus landscape type not only directly affects the psychological recovery of college students, but also can produce the effect of promoting psychological recovery through the interaction between the perception of campus landscape type and pro-social behavior. The mediating effect of pro-social behaviors accounted for 41.51% of the total effect, and the effect was relatively obvious, with the social reciprocity factor having the greatest influence.

There are also deficiencies in this study. First, the research data used in the article mainly comes from questionnaire survey, which is cross-sectional in nature and lacks continuous follow-up of the research subjects; continuous follow-up method can be used in future research, which allows us to observe the dynamic relationship between pro-social behavior in green space perception and psychological recovery. Second, the questionnaire survey method has limited measurements of environmental characteristics in space, and the design level characteristics of green space are not sufficiently described in the text; the design level characteristics have an important impact on the frequency, duration, and preference of green space visits; experimental observation methods, post-use evaluation, and spatial statistical analysis can be used to improve the spatial characteristics of green space in future studies. Third, the driving and influencing factors of college students’ pro-social behavior, green space perception, and psychological recovery are multifaceted, and in the future, more in-depth investigation and research can be done from more angles to further improve the research results.

## Data Availability

The original contributions presented in the study are included in the article/supplementary material, further inquiries can be directed to the corresponding author.
